# Comparative Analysis of Hemagglutination Inhibition and Plaque Reduction Neutralization Tests for Japanese Encephalitis Virus Antibody Detection

**DOI:** 10.3390/v17010104

**Published:** 2025-01-14

**Authors:** Cui Li, Jianqing Wan, Deli Wang, Lu Xiao, Xuni Li, Cunshuai Zhang, Zhao Wang

**Affiliations:** 1 China Institute of Veterinary Drug Control, Beijing 100081, China; licuigh@sohu.com (C.L.); wanjianqing126@126.com (J.W.); wdl567890@126.com (D.W.); xiaolu2046@163.com (L.X.); lxn20062006@126.com (X.L.); 2 School of Laboratory Animal & Shandong Laboratory Animal Center, Shandong First Medical University, Jinan 250117, China

**Keywords:** Japanese encephalitis, PRNT, hemagglutination inhibition, antibody

## Abstract

Japanese encephalitis (JE) is a zoonotic disease caused by the Japanese encephalitis virus (JEV), belonging to the *Flaviviridae* family. Diagnosis of Japanese encephalitis (JE) based on clinical signs alone is challenging due to the high proportion of subclinical cases. The Plaque Reduction Neutralization Test (PRNT) is considered the gold standard for detecting JE-specific antibodies because of its high specificity. However, PRNT is complex, time-consuming, and requires live viruses, limiting its applicability in routine diagnostics. In this study, we compared the sensitivity and correlation of the Hemagglutination Inhibition (HI) assay and PRNT for detecting JE antibodies in avian serum samples. We conducted a comparative analysis of the outcomes obtained from the PRNT and HI using 240 serum samples collected from 30 JEV-immunized avian subjects at various time points. Comparative analysis revealed a significant correlation between the HI and PRNT (R^2^ = 0.9321, *p* ≤ 0.0001). The Bland–Altman analysis also exhibited favorable concordance between the two assays. Consequently, HI may function as a viable substitute for PRNT in the screening of a substantial number of serum samples.

## 1. Instruction

Japanese encephalitis (JE), also known as epidemic B encephalitis, is a zoonotic disease caused by the Japanese encephalitis virus (JEV) of the *Flaviviridae* family. It infects a variety of animals such as humans, pigs, horses, and rats, and is transmitted by mosquitoes [1,2]. JEV is a single-stranded, positive-sense RNA virus with an 11 kb genome [3]. JE is extremely harmful to humans and causes 70,000 human cases, including 15,000 deaths annually. The *Culex* mosquito is the primary vector of JEV transmission [2,4]. Although infection in adult swine does not typically cause symptomatic disease, it can lead to abortion, stillbirths, and birth defects, causing notable reproductive problems [4,5]. Equines are susceptible to infection and may develop neurological symptoms [6].

JE poses a significant threat to human health and the swine industry. In the absence of a specific therapeutic agent for JE, prophylactic vaccination emerges as a pivotal strategy for disease prevention and control [7,8]. In China, a live-attenuated vaccine (SA14-14-2) has been approved for swine, whereas an inactivated vaccine (P3 strain) is authorized for human administration. Monitoring antibodies in susceptible animal populations is crucial. JE infection is typically confirmed by detecting virus-specific antibodies in the cerebrospinal fluid (CSF) or serum.

The Plaque Reduction Neutralization Test (PRNT) is the gold standard for detecting JEV. The principle of PRNT involves incubating serum samples with a live virus to allow neutralizing antibodies to bind to the virus. The mixture is then added to cell cultures, where plaques (areas of infected cells) are quantified. The degree of plaque reduction reflects the presence and titer of neutralizing antibodies, providing a direct measure of immune response. However, relying on the short period of viremia makes viral genome detection unreliable [9,10]. The PRNT is technically demanding and requires live JEVs, limiting its clinical application. Numerous serological assays have been developed and are widely recommended for diagnosing Japanese encephalitis (JE) in animals. According to the World Organisation for Animal Health (WOAH), these include the Plaque Reduction Neutralization Test (PRNT), Hemagglutination Inhibition (HI) test, enzyme-linked immunosorbent assay (ELISA), and complement fixation (CF) test [11,12,13,14]. Each assay has unique strengths and limitations, depending on the diagnostic requirements and available resources. Virus isolation and RT-PCR demonstrated insensitivity, owing to the low level of viremia at the time of clinical symptom recognition.

The HI test is a classical serological method used to detect antibodies by measuring the ability of serum antibodies to prevent viral hemagglutinin from agglutinating red blood cells. This test is widely employed because of its simplicity, cost-effectiveness, and ability to process large numbers of samples rapid [15] to detect and titrate various viruses, including influenza virus and Newcastle disease virus. Compared to the PRNT, the HI assay offers technical simplicity, speed, and cost-effectiveness. Although the HI assay is widely used to detect antibodies against various viruses, its application to JEVs is less common. Previous studies have explored the use of an HI assay for JEVs, demonstrating its potential as a valuable tool in virological research and public health surveillance [16]. Additionally, comparisons with PRNT have shown that while PRNT is the gold standard for its high specificity, the HI assay offers practical advantages in simplicity and scalability for high-throughput applications.

The PRNT and HI assay for detecting JEV antibodies have not been comprehensively validated or systematically compared. Moreover, there are few studies that specifically investigate the correlation between the outcomes of these two methods. Therefore, we conducted a comparative analysis between the HI and PRNT assays, using 240 avian serum samples collected at various time points from avians immunized with JE vaccines (SA-14-14-2 and P3 strains). This evaluation aims to ascertain the potential of HI as a standardized assay for JEV detection.

## 2. Material and Methods

### 2.1. Cell Culture and Virus

The cell line BHK-21 was cultured at 37 °C in 5% CO_2_ in RPMI-1640 (Gibco, Big Cabin, OK, USA) supplemented with 10% fetal bovine serum and 1% (100 U/mL) penicillin and (100 μg/mL) streptomycin. The JE live-attenuated vaccine (SA-14-2 strain) and inactivated vaccine (P3 strain) used in this study were stored at the China Institute of Veterinary Drug Control.

### 2.2. Reagents

HI test agents for the Japanese Encephalitis virus were purchased from the China National Institute of Viral Disease Control and Prevention. In addition, 25% kaolin suspension, 0.4% egg white PBS, 40% goose erythrocytes, and 0.5% goose erythrocytes were sourced as before [17].

### 2.3. Experimental Immunization and JE-Positive Serum

Thirty 30 days-old specific pathogen-free (SPF) chickens were used in this experiment. The chicken received four vaccine shots: two with the live-attenuated SA14-14-2 and two with the inactivated P3 strain, spaced two weeks apart. The first involved intranasal administration of 3 mL live-attenuated vaccine (SA14-14-2 strain), followed by two subsequent intramuscular injections, also two weeks apart, with the inactivated vaccine (P3 strain). Serum samples were collected before the 1st, 2nd, 3rd, and 4th dose of vaccination, as well as 7 d after the 2nd, 3rd, and 4th dose of vaccination, and finally, 14 d after the 4th vaccination. In total, 240 blood samples were collected from 30 specific pathogen-free chickens. Blood samples were centrifuged at 2500 rpm/m for 15 min to collect the serum. All the sera were stored at −20 °C and used to compare the results obtained for the HI and PRNT.

### 2.4. HI Assay

The HI assay was conducted according to the World Health Organization (WHO) standard procedure [18]. Serum (100 μL) was mixed with PBS (400 μL) and the 25% kaolin solution (500 μL). The serum mixture was inactivated at 60 °C for 30 min followed by adding 100 μL of 0.5% goose erythrocytes resulting in an initial testing dilution of 1:10. And then, the mixture was incubated at 37 °C for 30 min with shaking 4 times and centrifuging at 2000 rpm/m for 10 min. The supernatant was collected and used as serum. Virus suspensions (25 μL) containing the 10-time diluted JE virus antigen (strain SA14-14-2, 100 PFU/0.1 mL) were incubated for 1 h with 25 μL serial 2-fold dilutions of antiserum. Following incubation, 50 µL of standardized goose RBCs was added to all wells. Plates were observed for agglutination after one hour of tilting at an angle of 45–60°. The reciprocal of the highest dilution of serum or plasma that completely inhibited hemagglutination was determined as the HI titer [18]. HI antibody titers ≥ 1:10 were considered positive.

### 2.5. PRNT

Serum samples were inactivated for 30 min at 56 °C in a water bath and serially diluted two-fold up to 1:6400 in RPMI-1640 (GIBCO, Big Cabin, OK, USA). The serum examined at different dilutions was mixed with 100 PFU/0.1 mL of JE virus antigen (strain SA14-14-2) and incubated in a water bath at 37 °C for 90 min with shaking at every 30 min interval. All the neutralized viral solutions were inoculated in 6-well plates filled with BHK21 cell monolayers and were kept for 90 min at 37 °C in a 5% CO_2_ incubator. The supernatant was discarded and washed three times with PBS. Next, the RPMI 1640 medium (0.5 mL) containing 4% fetal bovine serum mixed with 2% low melting point agarose was added into each well. The 6-well plates were incubated at 37 °C in a 5% CO_2_ incubator for 5 days, after which the cells were fixed with 4% paraformaldehyde and stained with a 1% crystal violet solution. In the virus regression control, wells with 100 PFU of the virus should show approximately 100 plaques, and wells containing 0.1 PFU of the virus should show no plaques; both cell and serum control wells should show no plaques. The PRNT_50_ titer was chosen according to a WHO guideline [19].

### 2.6. Statistical Analysis

All assays were independently repeated at least three times in triplicate. Statistical analyses were performed using GraphPad Prism v.9.4. The correlation between two assays was analyzed via linear regression using Spearman’s correlation coefficient with a two-tailed *p*-value test and a 95% confidence interval. Correlations between the PRNT and HI were plotted with a 95% confidence interval. A Bland–Altman analysis was used to analyze the agreement between the titers obtained by the HI and PRNT assays.

## 3. Results

### 3.1. Comparison of HI and PRNT Assay Antibody Titers

The HI and PRNT assays were performed on 240 JE-positive serum samples from the SA14-14-2 and P3 strains of immunized chickens from eight different time points (before first vaccination, before second vaccination, 7 d post-second vaccination, before third vaccination, 7 d post-third vaccination, before fourth vaccination, 7 d post-fourth vaccination, and 14 d post-fourth vaccination). All serum samples were tested in triplicate with standard samples (positive and negative serum) on different days in the same assay. We explored the relationship between the HI and PRNT assays using the same serum samples in both experiments.

**Figure 1:**
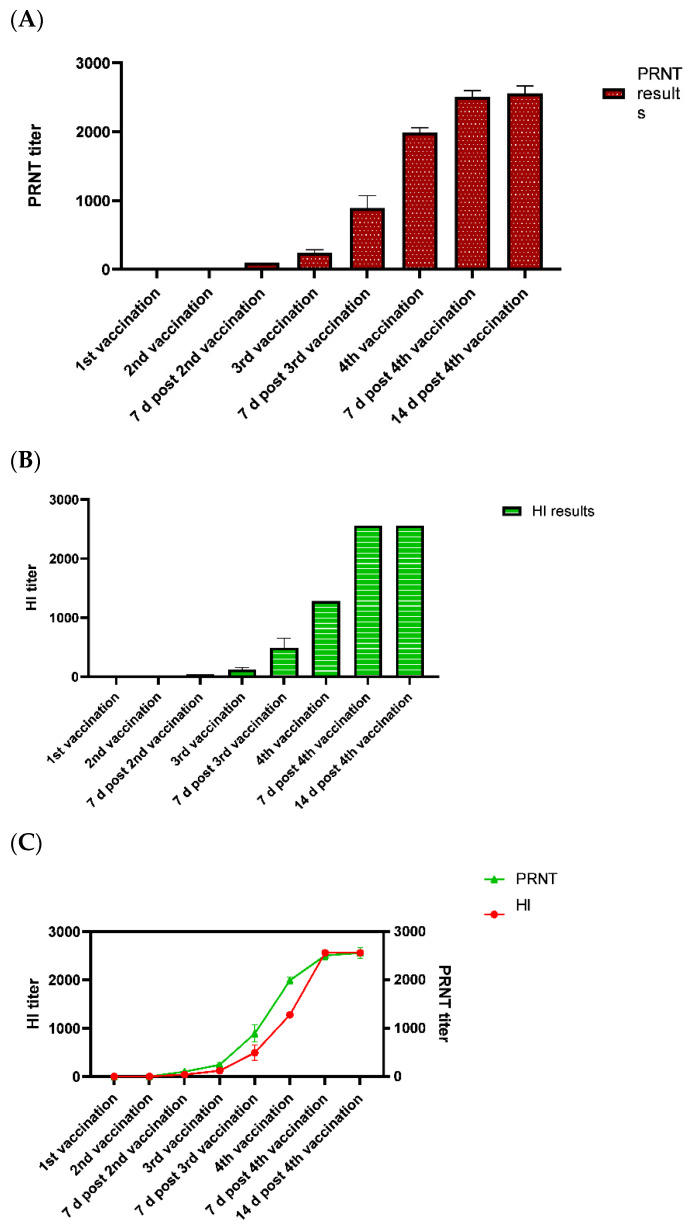
Comparison of the JE antibody titer in 240 serum samples as detected by HI and the PRNT against the JE agent (SA-14-14-2). (**A**) JE antibody titers were measured using the PRNT assay in avian serum samples collected at eight different time points: before 1st vaccination, before 2nd vaccination, 7 d post-2nd vaccination, before 3rd vaccination, 7 d post-3rd vaccination, before 4th vaccination, 7 d post-4th vaccination, and 14 d post-4th vaccination. (**B**) JE antibody titers were measured using the HI assay in the same avian serum samples collected at the aforementioned eight different time points. (**C**) Linearity comparison of the HI and PRNT assays for JE antibody detection at the eight different time points, illustrating the consistent performance across both methods.

All neutralizing antibody titers were set to endpoint values, following standard practices in serological assays, as described by Perng et al. [20]. The findings revealed that as the PRNT titer decreased below 1:100, the corresponding HI titer also decreased to levels below 1:40. Within the PRNT range of 1:100 to 1:400, the corresponding HI titers varied between 1:80 and 1:160. In the PRNT range of 1:400 to 1:1500, the HI titers ranged from 1:320 to 1:640. When the PRNT titer was approximately 1:2000, the corresponding HI titer was between 1:1280 and 1:2560 (Figure 1).

### 3.2. Nonlinear Regression Analyses of HI and PRNT

**Figure 2:**
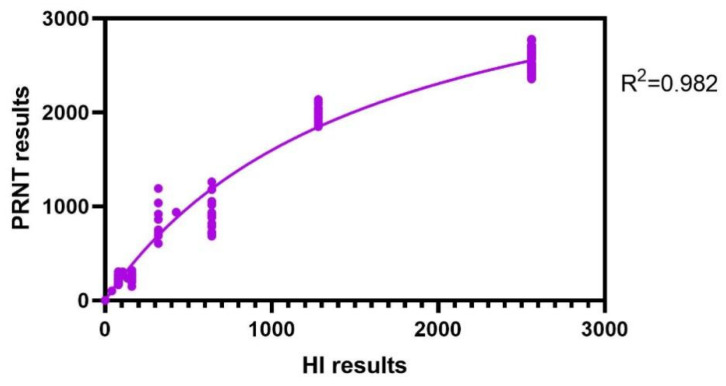
Correlation and Michaelis–Menten nonlinear regression analysis between PRNT and HI titers of the JE antibody in 240 serum samples. The nonlinear regression analysis of the dataset was performed to check the regression. The X line shows an average of three independent HI results at eight different time points: before 1st vaccination, before 2nd vaccination, 7 d post-2nd vaccination, before 3rd vaccination, 7 d post-3rd vaccination, before 4th vaccination, 7 d post-4th vaccination, and 14 d post-4th vaccination. The Y line shows an average of three independent PRNT results at the aforementioned eight different time points. The coefficient of determination (R^2^) was 0.982.

The nonlinear regression analysis was conducted to determine the relationship between HI and the PRNT (Figure 2). The coefficient of determination (R^2^) was 0.982. This high R^2^ value indicates a strong correlation between the HI and PRNT assays, suggesting that the HI assay can reliably reflect the results obtained from the PRNT assay. HI and the PRNT showed high agreement.

**Figure 3:**
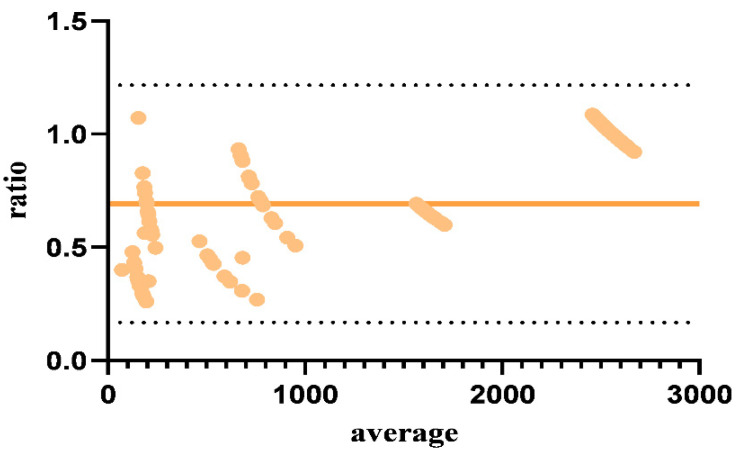
Bland–Altman plots for serum-titrated plasma samples. Bland–Altman plots were created as a graphical representation of agreement between the PRNT and HI assays by plotting the differences in the log 10 titers between measurements obtained from the two assays for a ratio (*y* axis) against the average (*x* axis). The red line shows that the bias is 0.6922, and dotted lines show the results of the 95% limits of agreement (1.96 SDs).

Bland–Altman analysis was used to assess the degree of agreement between the two assays. This method uses a straightforward graphical technique in which the ratio of the results from two assays is plotted against the average of the same readings. The analysis demonstrated a bias of 0.6922 with a standard deviation (SD) of 0.2675. This indicates that, on average, the ratio of the results from the two assays deviated from 1 by 0.6922 units, with most ratios falling within + 0.535 (mean ratio + 1.96 SDs). The high level of agreement shown by these values supports the reliability of the Hl assay compared to the PRNT assay (Figure 3).

## 4. Discussion

The detection of antibodies against JEV is crucial for both epidemiological surveillance and evaluation of vaccine efficacy. Among the various serological methods available, the PRNT is regarded as the gold standard because of its high specificity and sensitivity in measuring neutralizing antibodies. However, the PRNT method is inherently time-consuming, technically demanding, and requires live viruses, which limits its practical application in routine diagnostics and large-scale studies.

Several methods have shown promising alternatives to the PRNT for the detection of JEV antibodies, albeit with certain limitations. ELISA, particularly the IgG ELISA and antigen capture ELISA, offer simpler, rapid, and cost-effective alternatives, with the latter being highly specific because of the use of monoclonal and polyclonal antibodies targeting the JEV protein [21,22]. However, the sensitivity of ELISA is lower than that of the PRNT and may not always distinguish between JEV and other flavivirus antibodies [22].

RT-qPCR is valuable for detecting viral RNA during acute infections, providing rapid and sensitive results. It is highly sensitive and specific for JEV detection, particularly in early infection stages. While our study focused on serological methods, the timing of sample collection remains crucial for accurate antibody and viral RNA detection. However, its effectiveness is limited to periods of active viremia and may miss infections detected through serological methods [21]. Integrating RT-PCR with serological assays could enhance diagnostic strategies. The Focus Reduction Neutralization Test (FRNT) offers a quicker, less labor-intensive alternative to PRNT; however, it requires the handling of live viruses and specialized expertise [21]. An Indirect Immunofluorescence Assay (IFA), although reliable, is complex and requires specialized equipment and expertise, limiting its practicality for large-scale use [22].

Conversely, the HI assay is a simpler, faster, and more cost-effective alternative. The HI assay is well suited for high-throughput applications, as evidenced by its successful use in 96-well plate formats in previous studies, enabling simultaneous processing of large numbers of samples with high efficiency and reproducibility. This adaptability makes it particularly valuable for large-scale surveillance and diagnostic programs. Our comparative analysis of the HI and PRNT assays using 240 serum samples from avian subjects immunized with live and inactivated JEV vaccines demonstrated a significant correlation between the two methods. In this study, we used intranasal inoculation to administer the JEV vaccine. This method was selected based on evidence suggesting that intranasal administration can enhance mucosal immunity and provide the first line of defense against pathogens entering the respiratory tract. Previous studies have shown that intranasal vaccination can induce both systemic and mucosal immune responses, crucial for comprehensive protection against viral infections. While licensed JEV vaccines are administered subcutaneously, intranasal inoculation offers a non-invasive alternative that can be particularly beneficial for targeting respiratory pathogens [15]. Specifically, the strong R^2^ and robust statistical validation from nonlinear regression results also indicate that HI showed a high degree of agreement with the PRNT assay. Additionally, the Bland–Altman analysis showed a high level of agreement between the HI and PRNT results, supporting the potential of the HI assay as a viable alternative to the PRNT. The sensitivity and specificity of the HI assay indicate that while PRNT remains highly sensitive, the HI assay offers comparable specificity, highlighting its potential utility in diagnostic settings.

This study exclusively examined avian serum samples. Further research involving human serum samples is required to validate the applicability of HI assays across different species. Related work, such as screening avian serum from farms and not SPF chickens to compare the relationship between HI and PRNT assays, will be our future research direction. Nevertheless, our findings suggest that the HI assay can serve as a practical tool for large-scale serological surveys and vaccine efficacy studies, offering a balance between accuracy and feasibility. We recognize that our study, using only SPF chickens, is limited to the generalizability of our findings. We acknowledge that our study does not fully align with the WOAH-recommended HI test protocol, particularly in terms of serum preparation steps such as acetone or kaolin treatment to remove nonspecific inhibitors. Additionally, the focus of our study on avian serum samples limits its direct applicability to the broader host range recommended by WOAH, such as pigs and horses. The potential for false positives in animals exposed to other viruses and false negatives due to serological differences in JEV genotypes are important considerations. In this study, we did not evaluate the cross-reactivity of the HI assay with other flaviviruses, such as the West Nile virus, due to the unavailability of these viruses in our region. This limitation highlights the need for future studies to assess the specificity of the assay in broader diagnostic contexts.

We aimed to evaluate the specificity of the HI test in housed animals such as swine and plan to assess the detection of antibodies against various JEV genotypes in future studies.

In conclusion, the HI assay is a promising alternative to the PRNT for detecting JEV antibodies, particularly in settings where high throughput and rapid turnaround are required. Future studies should focus on optimizing the HI protocol and exploring its application in diverse epidemiological contexts to completely establish its utility for JEV surveillance and control efforts.

## Data Availability

The original contributions presented in this study are included in the article. Further inquiries can be directed to the corresponding authors.
